# Association between gut microbiota, microbial network, and immunity in pregnancy with a focus on specific bacterial clusters

**DOI:** 10.3389/fmicb.2023.1314257

**Published:** 2023-12-13

**Authors:** Hao Yan, Xinyuan Liang, Huijuan Luo, Xiaomei Tang, Xiaomin Xiao

**Affiliations:** ^1^Department of Obstetrics and Gynecology, The First Affiliated Hospital of Jinan University, Guangzhou, China; ^2^Department of Obstetrics, The Second Clinical Medical College (Shenzhen People's Hospital), Jinan University, Shenzhen, China

**Keywords:** gut microbiota, pregnancy, cluster analysis, microbial network, immunity

## Abstract

**Background:**

The community characteristics of the gut microbiota are not well defined and are not as widely studied as the functions of individual bacteria. This study aims to investigate the community composition of intestinal flora in women of childbearing age by conducting cluster analysis of gut microbiota and analyzing the relationship between different clusters and immune status.

**Methods:**

A total of 45 women of childbearing age were recruited in the study, including 15 non-pregnant women and 30 women in late pregnancy, and stool samples were collected twice during the third trimester, specifically at 32 weeks and at full term. The gut microbiota data was analyzed using 16S rRNA amplicon sequencing. Partitioning Around Medoids algorithm was employed to assess microbial clustering patterns. Microbial network for each cluster was performed and plasm cytokines were measured to analyze the relationship between specific genera and immune state in clusters.

**Results:**

There were three distinct clusters of intestinal community composition in women of childbearing age. Cluster 1 (PAM_1) was characterized by a high abundance of *Bacteroides*, while cluster 2 (PAM_2) showed higher levels of *Bifidobacterium* and *Blautia*, along with a significantly increased Firmicutes to Bacteroidota ratio. Cluster 3 (PAM_3) displayed a high abundance of *Escherichia-shigella*. PAM_1 was the most dominant cluster in non-pregnant women, and this dominant cluster was also one of the main in late pregnancy. At full term, the majority of subjects retained the same cluster as at 32 weeks, while a few experienced a shift. The microbial correlation networks differed across the three clusters, with PAM_1 exhibiting higher modularity and fewer connections. Analysis of the correlation between genera and plasma cytokines showed significant differences in their associations with cytokines between pregnancy and nonpregnancy within the same cluster, and the same genera had different effects in different clusters.

**Conclusion:**

Women of childbearing age exhibit three distribution patterns of gut microbiota, and the intestinal clusters reshaped during late pregnancy in a small population. Different clusters may have diverse immunomodulatory effects in different physiological states. When studying the gut microbiome during pregnancy, it is crucial to consider the cluster differences within healthy women.

## Introduction

The gut microbiota, which consists of symbiotic microorganisms within the human body, is intricately linked to host physiology and pathophysiology (de Vos et al., 2022), and can be influenced by environmental, dietary, and host factors (Dong and Gupta, 2019). The maternal microbiome is thought to have a significant impact on both normal pregnancy maintenance and the health of offspring (EbioMedicine, 2021). Therefore, gaining a comprehensive understanding of the gut microbiota during pregnancy is of great importance. Previous studies have reported changes in the diversity of maternal intestinal flora during the third trimester, as well as variations in the abundance of Proteobacteria and Actinobacteria (Koren et al., 2012), which may be associated with physiological shifts such as metabolic changes during pregnancy. It has also been observed that the intestinal flora remains relatively stable throughout pregnancy (DiGiulio et al., 2015). However, further research is still needed to verify the composition of the intestinal flora in women during normal pregnancy. Pregnancy is a unique period characterized by significant changes in maternal immunity and metabolism as the fetus grows and develops (Zakaria et al., 2022). The maternal immune system is naturally suppressed during the second and third trimesters to support fetal growth until labor is initiated (Menon, 2022; True et al., 2022). The intestinal microbiota and its metabolites play a regulatory role in both local and systemic immune responses (Schluter et al., 2020; Wastyk et al., 2021), and alterations in immune status during pregnancy may be correlated with maternal gut microbiota.

The notion of “enterotype,” which was introduced in 2011, suggests that the gut microbiota can be categorized into several distinct microbial community patterns within the population, and these enterotypes are associated with functional differences (Arumugam et al., 2011). Recent studies utilizing unsupervised clustering methods have also revealed potential patterns in the human gut microbiome (Tap et al., 2023). In a study focused on puerperal gut microbiota from the Spanish-Mediterranean region, an enterotype cluster analysis demonstrated that the maternal microbiota can be divided into two distinct intestinal clusters characterized by *Prevotella* (cluster I) and *Ruminococcus* (cluster II), respectively (Garcia-Mantrana et al., 2020). Additionally, an investigation into the intestinal microbiota of sows throughout various stages of gestation revealed different community patterns during late gestation compared to the early gestation period (Berry et al., 2021). Significant variations exist in the gut microbiota composition among individuals, and the differences in gut microbiota communities in women of childbearing age, particularly during pregnancy, are still not fully understood. Therefore, this study focuses on the community clustering of gut microbiota, as a pilot to preliminarily explore the changes in community composition, structural characteristics of microbial networks, and the potential relationships with the immune status of childbearing age women.

## Materials and methods

### Study design and sample collection

In this study, we recruited locally residing healthy non-pregnant women of childbearing age and late-pregnant women who underwent labor and delivery at the First Affiliated Hospital of Jinan University. The inclusion criteria were: (1) Chinese women in their first pregnancy with a singleton fetus; (2) aged between 18 and 34 years; (3) no complications during pregnancy and normal pre-pregnancy body mass index (BMI) of 18.5 to 23.9 kg/m^2^; (4) living in Guangzhou, and receiving regular prenatal examinations at the hospital. Non-pregnant women were required regular menstrual cycles, and other of the recruitment criteria were the same as those for pregnant women (except for pregnancy). Exclusion criteria were: (1) previous history of gastrointestinal diseases and family history of related gastrointestinal diseases; (2) taking antibiotics, probiotics, and prebiotics during pregnancy (for non-pregnant subjects, a history of related medication use within the past 6 months); (3) with chronic disease prior to the current pregnancy.

A total of 30 women in the third trimester of pregnancy and 15 non-pregnant women of childbearing age were included in the study, and all subjects provided written informed consent. All pregnant women underwent regular prenatal check-ups, and two fecal samples were collected from each: one at 32 weeks of gestation and another at full-term before delivery. The method of collecting feces was to empty the bladder first to avoid the mixing of urine and vaginal secretions, collect the size of about 3cm^3^ volume of feces, and then use a sterile cotton swab to take 3–5 g of the internal substantial part of the feces into a specimen box, and then put it in the refrigerator at −80°C within half an hour. We would provide a unified new sampling pad sheet to collect feces. Pregnant women can defecate at home or in the hospital, but must be transferred to the −80°C refrigerator for storage within half an hour in a low temperature environment. For the control group, stool samples were collected once by the same method on days 7–14 of the menstrual cycle. Peripheral venous blood was collected on the day of fecal collection, and the plasma was stored in a −80°C refrigerator after centrifugation. Collect general information about the mother and baby. The non-pregnant subjects were the control group, and the pregnant participants were the pregnancy group. The pregnancy group consisted of a late pregnancy (PL) group at 32 gestational weeks and a full-term pregnancy (PT) group, with full-term pregnancy defined as the pregnancy after 37 weeks and before the onset of labor.

### DNA extraction and 16S rRNA sequencing

The DNA of samples was extracted by the CTAB/SDS method, and the purity and concentration of DNA were detected by agarose gel electrophoresis. Appropriate DNA samples were taken and diluted with sterile water to a concentration of 1 ng/μl. Using diluted DNA as a template, the selected V3–V4 variable region was amplified on a Bio-rad T100 gradient PCR apparatus using a specific primer 341F-806R with barcode and DNA polymerase. The libraries were constructed using the Illumina TruSeq^®^DNA PCR-Free library preparation kit, and then quantified and identified using qubit analysis. Finally, computer sequencing was performed using the NovaSeq6000 platform.

### Analysis of 16S rRNA sequencing data

Sample data were separated from offline data according to Barcode and primer sequences, and raw tag data were first spliced using FLASH software. According to the tags quality control process in QIIME (V1.9.1), the raw tags were further screened to obtain high-quality clean tag data, which were then compared with the species annotation database to remove the chimeric sequences and obtain the final effective tags. Using Uparse (v7.0.1001) to cluster effective tags into operation classification units (OTUs) with a similarity threshold of 97%. Classifications were assigned to OTUs based on the Silva database.

### Plasma cytokines testing

Plasma cytokines (interleukin [IL] IL-1β, IL-2, IL-6, IL-10, IL-12, interferon-γ, and tumor necrosis factor-α) levels were measured using the Luminex Discovery Assay Human Premixed Multi-Analyte Kit on Luminex^®^ 200^™^ System.

### Statistical analysis

Statistical analysis was conducted using R software and SPSS software. Measures were presented as mean ± standard deviation. *T*-tests and ANOVA were employed for group comparisons. Cluster analysis was performed using the Partitioning Around Medoids (PAM) algorithm based on the Jensen-Shannon distance. To identify differences in microbial characteristics between clusters, Linear Discriminant Analysis (LDA) Effect Size (LEfSe) (Segata et al., 2011) was utilized, with a threshold LDA value >4.0. Genera with an abundance >0.1% and present in more than 20% of the samples were selected for visualization of the microbial network using Cytoscape (Shannon et al., 2003). Criteria for network visualization included an absolute r-score > 0.6 and *p* < 0.05, as determined by Spearman correlation analysis. Heatmaps illustrating the correlation between genera and cytokines were generated using the pheatmap package in R.

## Results

A total of 15 healthy non-pregnant women and 30 women in late pregnancy were included in this study. All the women were of Han nationality and local resident in south China. There was similar demographic information among the enrolled subjects (Table 1). Finally, 75 fecal samples were sent for 16S rRNA sequencing.

**Table 1:** Clinical characteristics of enrolled participants in this study.

Characteristics	Control group	Pregnant group	*t*	*p*-value
	*N* = 15	*N* = 30		
Age (year)	26.40 ± 1.84	26.67 ± 2.15	0.410	0.684
Height (cm)	160.20 ± 5.43	160.78 ± 7.29	0.274	0.786
Pre-pregnant weight (kg)	50.35 ± 4.54	51.62 ± 6.78	0.654	0.517
Pre-pregnant BMI (kg/m^2^)	19.57 ± 0.67	19.90 ± 1.46	1.022	0.312

Comparisons using Student’s *t*-test.

BMI, Body Mass Index.

### Community distribution characteristics of gut microbiota

**Figure 1:**
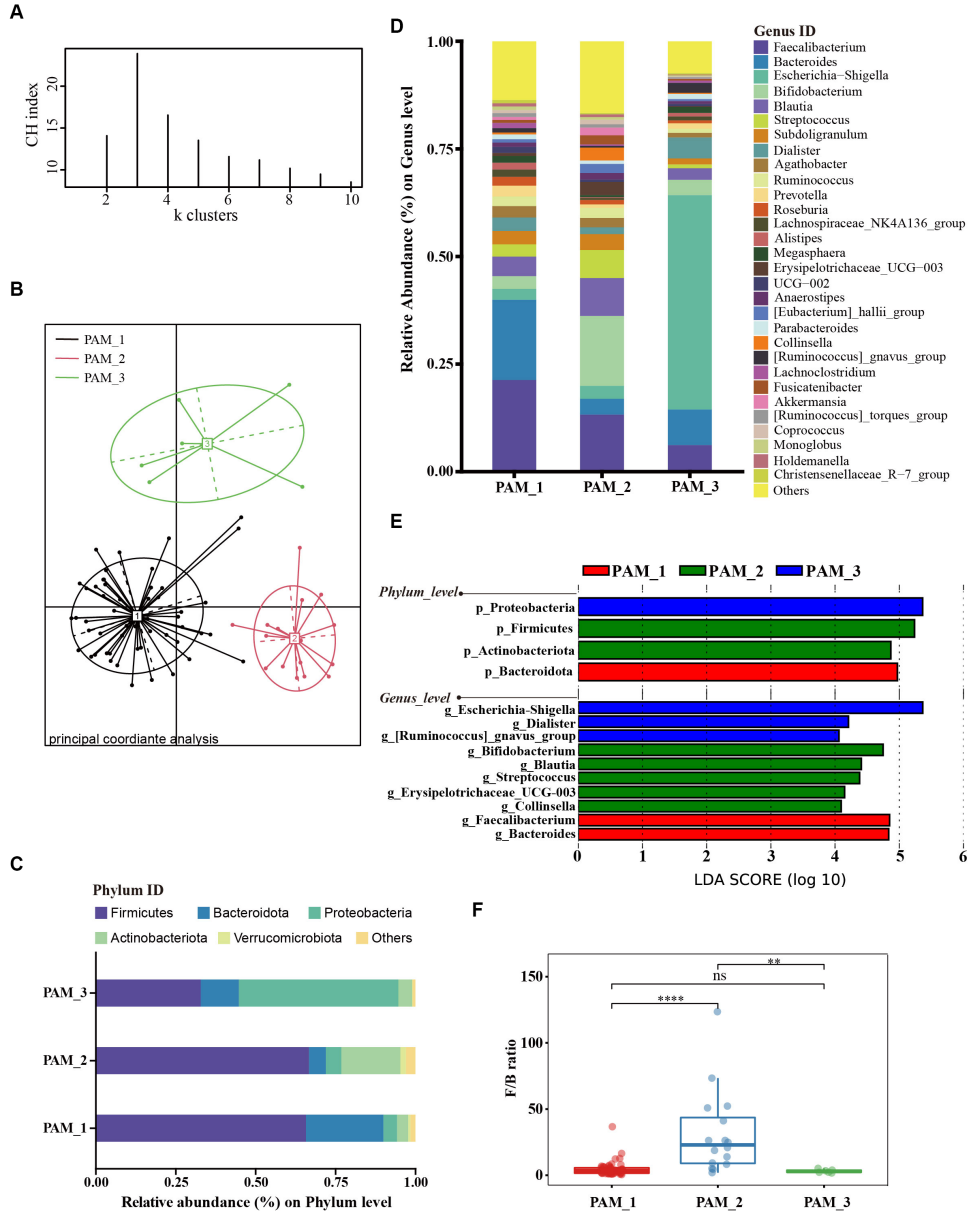
Cluster analysis of gut microbiota in women of childbearing age. **(A)** According to the abundance of dominant taxa, samples were clustered into the 3 clusters with the highest CH index by PAM algorithm; **(B)** Sample distribution based on Jensen-Shannon distance from three clusters; **(C)** The main phyla composition of the three clusters; **(D)** The composition of the top 30 genera in the three clusters; **(E)** LEfSe analysis at phylum and genus levels among three clusters (LDA score > 4.0, *p* < 0.05); **(F)** Difference in F/B ratio between three clusters.

To characterize the composition of the gut microbiota of pregnant and non-pregnant women, we performed cluster analysis of intestinal flora. The abundance distribution of the dominant taxa was quantified using the Jensen-Shannon distance. To determine the optimal number of groups, we calculated the Calinski-Harabasz (CH) index. According to the CH index (Figure 1A), the optimal number of clusters was determined to be three groups, and the Principal Coordinate Analysis (PCoA) demonstrated significant separation between the clusters after clustering (Figure 1B).

Upon analyzing the composition of the three clusters (Figure 1C), it was observed that cluster 1 (PAM_1) was predominantly composed of Firmicutes, followed by Bacteroidota, accounting for 65.48% ± 13.91 and 24.20% ± 13.21%, respectively (mean ± standard deviation). In cluster 2 (PAM_2), Firmicutes were also dominant, followed by Actinobacteriota, with relative abundances of 66.36% ± 12.60 and 18.45% ± 8.94%, respectively. Cluster 3 (PAM_3) showed a dominance of Proteobacteria (49.77% ± 17.20%) and a secondary presence of Firmicutes (32.71% ± 14.24%). At the genus level (Figures 1D,E), significant increases in abundance were observed in *Faecalibacterium* [phylum Firmicutes] and *Bacteroides* [phylum Bacteroidota] in PAM_1. PAM_2 exhibited notable increases in *Bifidobacterium* [phylum Actinobacteriota], *Blautia* [phylum Firmicutes], *Streptococcus* [phylum Firmicutes], *Erysipelotrichaceae_UCG-003* [phylum Firmiculus], and *Collinsella* [phylum Actinobacteriota]. PAM_3 was characterized by a significant increase in abundance of *Escherichia-shigella* [phylum Proteobacteria], while *Dialister* [phylum Firmicutes] and *[Ruminococcus]_gnavus_group* [phylum Firmicutes] also increased. The differential genera corresponded to the dominant phyla in each cluster, indicating variations in the abundance of dominant bacteria within different intestinal clusters. In addition, the Firmicutes to Bacteroidota ratio (F/B ratio) showed significant differences among the three clusters, with PAM_2 showing a significantly increased F/B ratio (Figure 1F).

### Changes in community clusters during pregnancy

**Figure 2:**
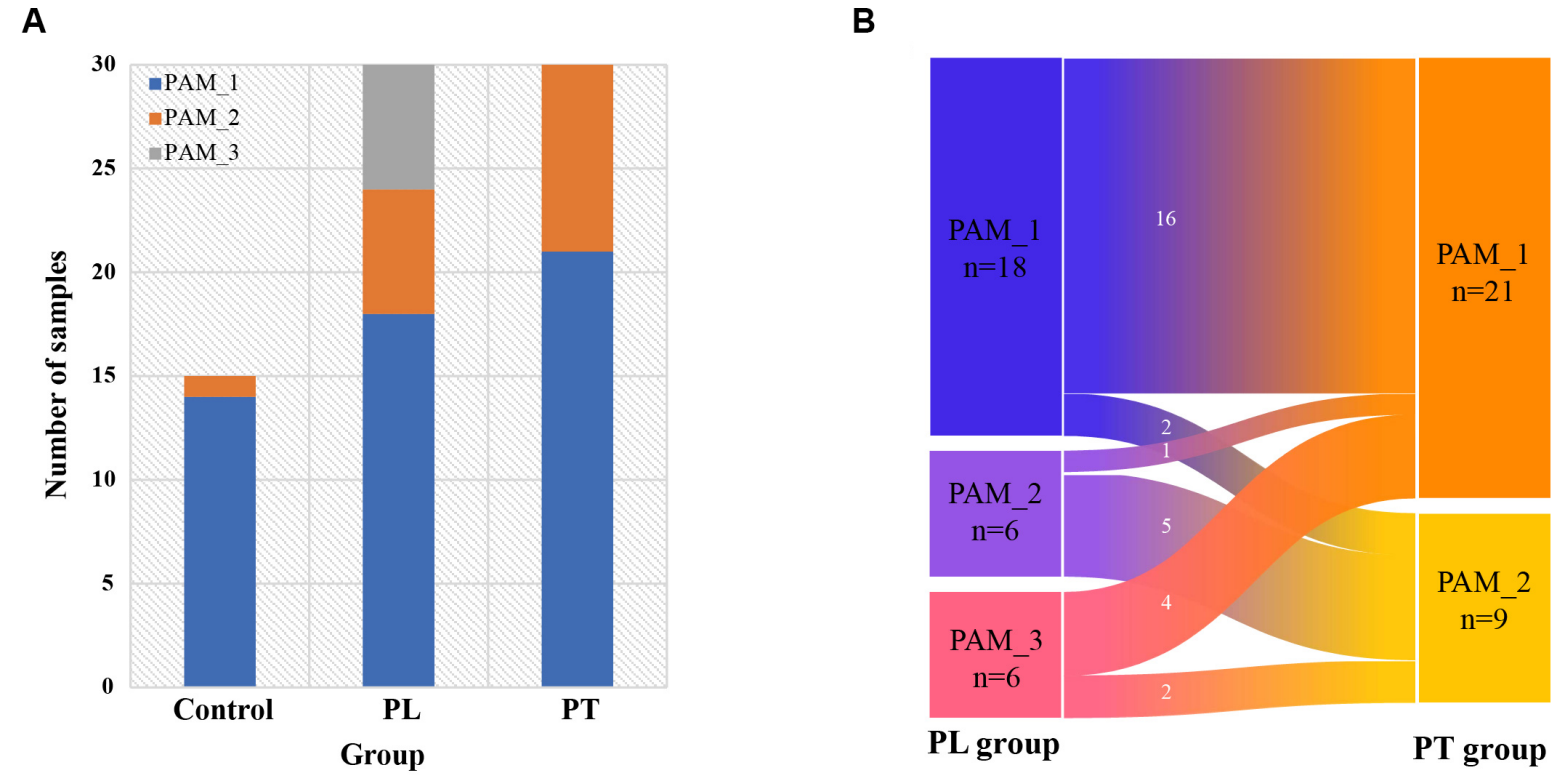
Intestinal cluster distribution characterization. **(A)** Composition of the three clusters in the control, PL, and PT groups; **(B)** Distribution of intestinal clusters in the pregnancy group of subjects from PL to PT period.

Based on the cluster distribution of gut microbiota in women of childbearing age (Figure 2A), it can be found that the main cluster was PAM_1, present in 14 out of 15 individuals in the control group, 18 out of 30 individuals in the PL group, and 21 out of 30 individuals in the PT group. Additionally, PAM_2 was predominantly found in the third trimester of pregnancy, with 6 out of 30 individuals in the PL group and 9 out of 30 individuals in the PT group, while being scarce in the control group (1 out of 15 individuals). Moreover, PAM_3 was exclusively observed in the PL group, accounting for a certain proportion (6 out of 30 individuals). These findings highlight the complexity of the gut microbiome at 32 weeks of pregnancy, with significant variations in the dominant taxa abundance within the population.

We conducted further analysis on the cluster changes within the same subjects from PL to PT (Figure 2B). The results revealed that the gut clusters remained consistent in the majority of subjects from 32 weeks to full-term pregnancy (21 out of 30 individuals, with 16 classified as PAM_1 and 5 as PAM_2). However, a small subset of individuals (9 out of 30) exhibited changes in their cluster communities. These findings suggest that during late pregnancy, the intestinal community remains relatively stable for most individuals, but a few individuals experience changes in their cluster communities during this period.

### Characterization of microbial networks of different clusters

**Figure 3:**
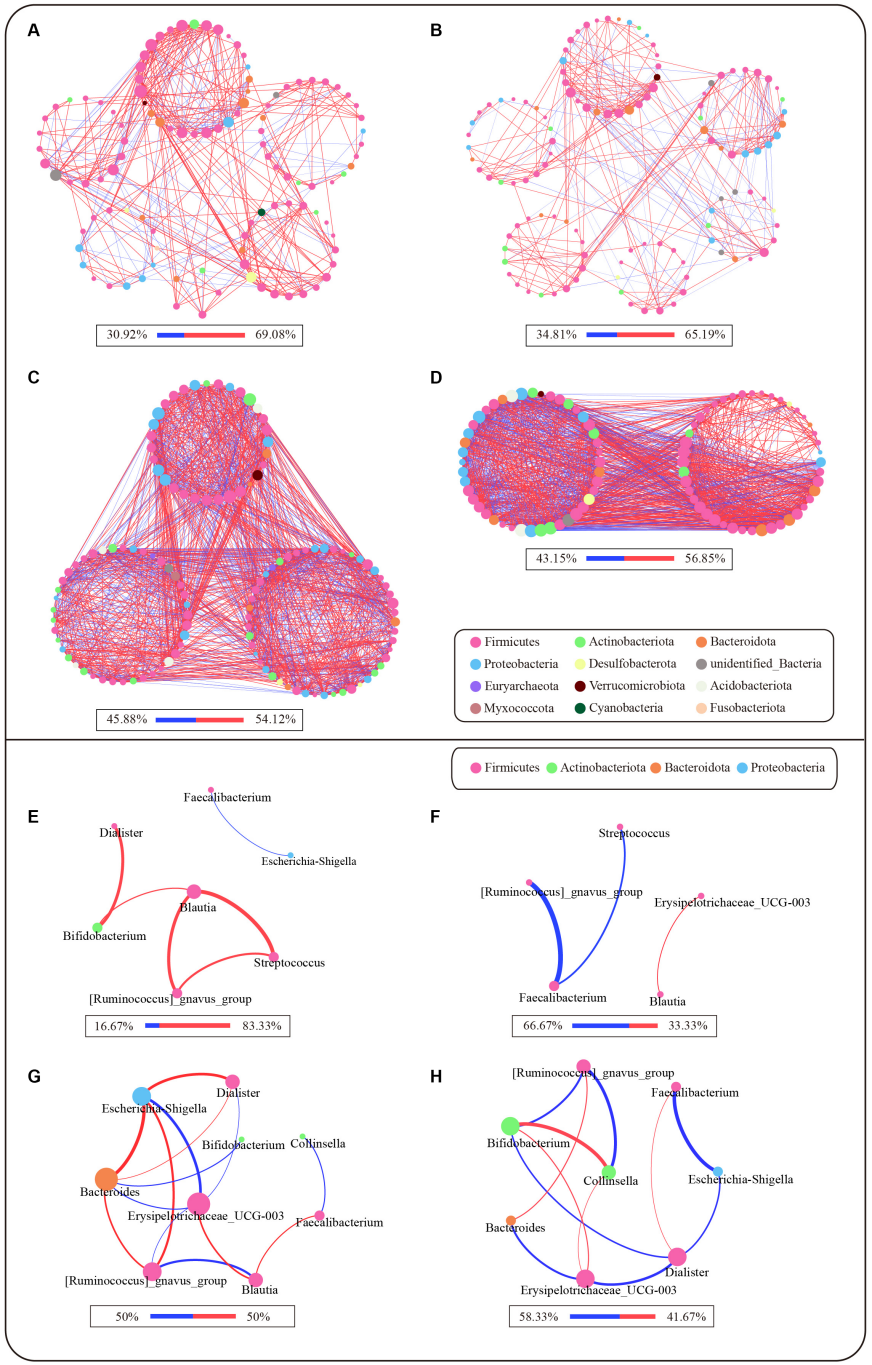
Microbial network of different clusters at genus level. The network was based on the spearman correlation analysis (*r* ≥ 0.6 and *p* < 0.05). Each node represents a genus in the network, and the color is indicated by its phylum level. The larger the nodes, the greater the number of correlation relationships in the network. The red lines are positive correlations, and the blue lines are negative correlations, and the proportion of positive and negative correlations is shown below each diagram. The larger the correlation coefficient r-score is, the thicker the lines are. **(A–D)** Microbial correlation networks between genera that satisfy abundance greater than 0.1% and have expression in more than 20% of samples for each cluster. **(A)** Network of PAM_1 in control group; **(B)** Network of PAM_1 in PL group; **(C)** Network of PAM_2 in PL group; **(D)** Network of PAM_3 in PL group. **(E–H)** Microbial correlation networks of differential genera selected from LEfSe in different clusters. **(E)** Differential genera network of PAM_1 in control group; **(F)** Differential genera network of PAM_1 in PL group; **(G)** Differential genera network of PAM_2 in PL group; **(H)** Differential genera network of PAM_3 in PL group.

We compared the correlation networks at the genus level between the control and PL groups for each cluster (Figures 3A–D). The results showed that, in comparison to PAM_2 and PAM_3, PAM_1 exhibited a sparser network with a higher degree of modularity. The number of correlations among genera was lower in PAM_1, with predominantly positive correlations. What’s more, the microbial networks became more complex in PAM_2 and PAM_3, with a decrease in modular structures, an increase in correlations among genera, and a higher proportion of negative correlations. The network attribute table (Table 2) further highlights differences between PAM_1, PAM_2, and PAM_3. In the table, the diameter is the maximum distance between any two nodes in the network; modularity measures the degree to which a network is divided into meaningful modules or communities; clustering coefficient measures the likelihood of a node being connected to its neighboring nodes; average degree measures the association between different nodes, and average path length is the average distance between pairs of nodes that have a connecting path in the network. PAM_1 exhibited a lower number of network nodes and edges compared to PAM_2 and PAM_3. The modularity coefficient was higher in PAM_1, indicating a more rational sub-network, while the clustering coefficient was lower, suggesting a more dispersed network among modules. Further observation of the correlation networks among differential genera (Figures 3E–H) showed that PAM_1 had fewer correlations, including fewer differential genera. Conversely, PAM_2 and PAM_3 exhibited relatively complex network relationships. Furthermore, it was found that the correlation between genera was not significantly associated with their abundance. For instance, *Bacteroides*, which was dominant in abundance in PAM_1, did not show a significant correlation with other differential genera, and *Escherichia-shigella*, which dominated the abundance in PAM_3, did not display an advantage in the number of network correlations.

**Table 2:** Microbial network topological coefficients of three clusters.

	Control	PL group		
	PAM_1	PAM_1	PAM_2	PAM_3
Number of edges	414	362	1759	1,541
Number of nodes	108	116	121	88
Diameter	4.73	5.29	1.98	2.24
Modularity	0.46	0.52	0.32	0.16
Clustering coefficient	0.41	0.37	0.54	0.69
Average degree	7.71	6.241	29.07	35.02
Average path length	2.02	2.22	1.21	1.13

### Correlation between gut cluster and plasm cytokines

**Figure 4:**
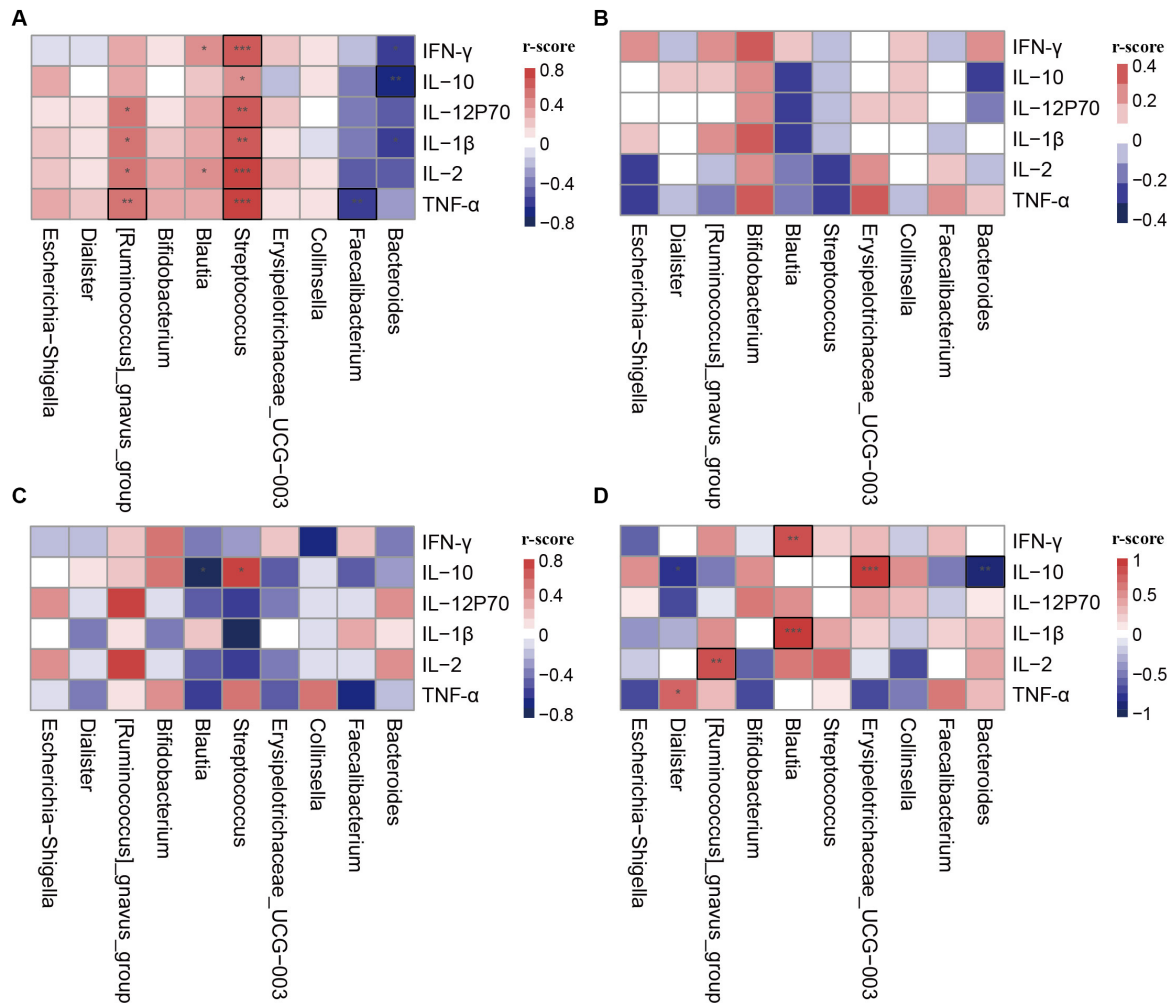
Correlation heatmap of cytokines and differential genera in different clusters. The differential genera were selected from LEfSe analysis. **(A)** PAM_1 in control group; **(B)** PAM_1 in PL group; **(C)** PAM_2 in PL group; **(D)** PAM_3 in PL group. *p < 0.1, **p < 0.05, ***p < 0.001, and those marked with a black box indicate statistical differences.

To investigate the function of different clusters, we analyzed the relationship between clusters and cytokines in different states. In late pregnancy, the cytokine levels were significantly lower compared to non-pregnancy, and there were no statistical differences observed between clusters in the PL group (Table 3). Correlation analysis was conducted between the differential genera and plasma cytokines. The results demonstrated that the correlation between clusters and cytokines in pregnant women significantly differed from that in non-pregnant women (Figures 4A,B). Moreover, the same genera showed different correlation trends in non-pregnancy and late pregnancy. For example, *Streptococcus* and *Blautia*, which exhibited positive correlations with cytokines in the PAM_1 cluster of the control group, displayed negative correlations in the PAM_1 cluster of the PL group. Similarly, *Faecalibacterium* and *Bacteroides*, which showed negative correlations in the PAM_1 cluster of the control group, did not exhibit significant correlations with the PAM_1 cluster in the PL group. During late pregnancy, slight differences were observed between the three clusters and cytokines (Figures 4B–D). In PAM_1, the genera generally displayed weak correlations with cytokines. The correlations between *Blautia* and cytokines in PAM_1 and PAM_2 were mostly negative, but predominantly positive in PAM_3. *Erysipelotrichaceae_UCG-003* exhibited a significant positive correlation with IL-10 in PAM_3, a negative correlation in PAM_2, and no significant correlation in PAM_1. These findings suggest that genera may not have a singular role, as they may play different immunomodulatory roles in different states and within different communities.

**Table 3:** Plasm cytokines level in different clusters.

	Control group	PL group		
	PAM_1 (*n* = 14)	PAM_1 (*n* = 18)	PAM_2 (*n* = 6)	PAM_3 (*n* = 6)
IFN-γ^#^	42.36 ± 23.68	15.19 ± 8.95	9.42 ± 3.47	8.49 ± 4.35
IL-10^#^	6.10 ± 2.69	1.87 ± 1.05	1.46 ± 0.56	1.87 ± 1.33
IL-12P70^#^	19.58 ± 8.71	7.14 ± 5.57	4.87 ± 2.50	4.71 ± 2.28
IL-1β^#^	11.51 ± 6.02	4.48 ± 2.50	3.21 ± 1.17	3.06 ± 1.35
IL-2^#^	12.35 ± 7.18	4.86 ± 2.48	4.20 ± 0.92	4.26 ± 0.95
TNF-α^#^	14.76 ± 5.87	7.28 ± 3.97	4.81 ± 2.34	4.74 ± 2.73

^#^Means a comparison of cytokine levels between PAM_1 of Control and PL groups, *p* < 0.05, using *t* test.

IFN-γ, interferon- γ; TNF-ɑ, tumor necrosis factor- α; IL, interleukin.

## Discussion

Through cluster analysis, we found differences in the community of the gut microbiota from non-pregnant to late pregnancy and full-term pregnancy in women. In late pregnancy, the gut microbiome exhibited a more complex composition, and the distribution of intestinal flora showed multiple distinct clusters. Additionally, different clusters displayed unique microbial networks. The correlation between gut genera and plasma cytokines differed among these distinct clusters, as well as within the same cluster under different physiological conditions, such as pregnancy and non-pregnancy.

In this study, we observed three distinct clusters in the abundance patterns of the dominant intestinal flora among women of childbearing age during late pregnancy and non-pregnancy. The primary intestinal cluster, labeled as PAM_1, was found to be dominant in women, characterized by Firmicutes and Bacteroidota as the predominant phyla. At the genus level, this cluster exhibited high abundance levels of *Faecalibacterium* and *Bacteroides*. These findings align with previous study reporting the abundance community of intestinal flora in the Chinese population (Lu et al., 2021). *Bacteroides* and *Faecalibacterium* are the core genera in the gut and have significant abundance advantages in healthy intestinal flora (Human Microbiome Project Consortium, 2012; Lopez-Siles et al., 2017). Notably, the participants in this study were long-term local residents and shared similar environmental factors and physical characteristics, including age and BMI. These factors, along with the strong association between microbiota variations and host geographical location as reported in previous research (He et al., 2018), may explain the predominance of the PAM_1 cluster in both pregnant and non-pregnant women.

The other two clusters were predominantly observed during late pregnancy, with PAM_2 being more abundant. In PAM_2, an increase in Actinobacteriota at the phylum level, commonly observed in the third trimester, could be a normal change in the gut microbiota during pregnancy (Koren et al., 2012; Li et al., 2023). The high abundance of *Bifidobacterium* in late pregnancy may also be influenced by progesterone, resulting in changes in its abundance (Nuriel-Ohayon et al., 2019). Late pregnancy is characterized by increased maternal glucose production and circulating lipids to support rapid fetal growth, accompanied by maternal insulin resistance (Lockitch, 1997; Bowman et al., 2021). The PAM_2 cluster, one of the major clusters in late pregnancy, demonstrated a significantly higher Firmicutes to Bacteroidota ratio. Previous research has suggested a close association between the Firmicutes to Bacteroidota ratio and metabolic diseases like obesity and insulin resistance (Magne et al., 2020). *Blautia*, as a potential probiotic, may play a role in regulating host health and alleviating metabolic syndrome, with its expression reported to be lower in women with gestational diabetes mellitus (GDM) than in healthy women (Ozato et al., 2019; Liu et al., 2021). The presence of *Erysipelotrichaceae_UCG-003* in PAM_2 was found to be associated with impaired glucose tolerance in the third trimester (Vavreckova et al., 2022). Changes in the abundance of these genera in PAM_2 may be linked to altered metabolic states during pregnancy and the maintenance of maternal health. The PAM_3 cluster was exclusively observed in women at 32 weeks of gestation. Previous research has observed an increase in Proteobacteria in some populations at T3 of gestation, and the transfer of the microbiome induced significant obesity and inflammation in sterile receptor mice (Koren et al., 2012). In a healthy and stable state, the relative abundance of Proteobacteria in the human gut could increase up to 45% (Caporaso et al., 2011), which may be a response to environmental changes (Shin et al., 2015). The expansion of Proteobacteria and *Enterobacteriaceae* reflects host energy imbalance and an unstable microbial community (dysbiosis) (Shin et al., 2015; Litvak et al., 2017). A study on maternal dyslipidemia during pregnancy found that clustering analysis of the intestinal microbiota, with dominant genera including *Faecalibacterium*, *Blautia*, and *Bacteroides*, showed higher levels of total cholesterol and triglycerides (Yang et al., 2023). These high-abundance genera were similar to that of cluster 1 and cluster 2 in this study, both of which were the most common community compositions among the late pregnancy population. The pregnant women included in this study were all normal pregnancies, and the clusters observed in the third trimester may be a result of the altered gut microbiota brought about by pregnancy and reflect the complex composition of the gut microbiome in late pregnancy. On the other hand, early colonizers in the neonatal gut include *Enterobacteriaceae* and *Bifidobacterium* and *Streptococcus*, and the increase of associated taxa in late pregnancy may also be required for the transfer of gut microbiota from mother to child (Xiao and Zhao, 2023).

The investigation of gut microbiota changes during pregnancy has been ongoing, with some studies identifying dynamic shifts in the gut microbiota, including an increase in Proteobacteria and a decrease in Bacteroidota (Koren et al., 2012; Li et al., 2023). However, other studies have reported a stable composition of the gut microbiota during pregnancy (Jost et al., 2014; DiGiulio et al., 2015). In this study, we tracked the cluster alterations of the subjects during late pregnancy and compared them with the clusters of non-pregnant women. Based on these findings, we propose that the gut microbiota of most women of childbearing age may generally maintain stability during late pregnancy, with a minority group experiencing pregnancy-induced changes. The remodeling of the intestinal flora is evident in the variations in abundance distribution among different taxa. Additionally, noticeable differences were observed in the network structure of microbial interactions among microorganisms. Similar cytokine levels predicted similar immune status among subjects with different gut clusters, so we hypothesize that the gut microbiota may be more holistic in character and that they work as a whole unit. It is possible that alterations in the composition of the gut microbiota are related to changes in the relationships among bacterial genera, because even the changed gut microbiota still maintains the host immunity balance.

Previous study has demonstrated that the gut microbiome may play a role in regulating host immunity (Blander et al., 2017). During pregnancy, maternal immunity undergoes complex and dynamic regulation to ensure fetal tolerance and protection (Bonney, 2016). While a pro-inflammatory response is involved in the implantation and delivery of a healthy pregnancy, a tolerant and anti-inflammatory environment is established to support normal fetal development (Yockey and Iwasaki, 2018). In this study, all subjects in the PL group were in the stage of labor not yet started, and significantly lower levels of cytokines were observed than those in the non-pregnant group, reflecting the immunosuppressive status of pregnancy. A study correlating maternal enterotypes with adipokines during labor and delivery found a significant association between the *Prevotella* enterotype and lipocalin levels (Rio-Aige et al., 2021). The different bacteria genera among three clusters were taken as characteristic bacteria genera to explore the relationship between different gut microbiota community and the immune status of the body. By correlating differential genera with cytokines, we found that the same genus had different effects on cytokines within different gut microbiota communities in late pregnancy. Previous exploration of microbial function has suggested that the effects of individual strains on host immunity can vary greatly depending on the context of the surrounding microbial community (Rice et al., 2022). Thus, the regulatory capacity of the gut microbiota may be exerted collectively by the entire community rather than by individual bacteria. Additionally, we observed that even within the same cluster, a single genus exhibited different immunomodulatory functions during pregnancy and non-pregnancy. We hypothesize that the function of the genus is closely related to the body’s immune status, and the functions of the genera are not uniform.

There were some limitations in this study that should be acknowledged. Firstly, the sample size was small, and a larger sample size would enhance the generalizability of the cluster analysis of intestinal communities. Secondly, the study lacked non-pregnant and post-pregnancy control observations for the same subjects, making it difficult to establish a comprehensive comparison. Additionally, it is worth considering that differences in enterotype may be influenced by dietary factors. Although the subjects in this study received balanced diet education during their antenatal examinations, the specific dietary structure was not thoroughly investigated, and the potential influence of dietary confounding factors cannot be excluded. Lastly, it is important to note that 16S amplicon sequencing provides accurate results only at the genus level, and further analysis of specific strains is not possible.

## Conclusion

In this study, we observed distinctive distribution patterns of gut microbiota in women of childbearing age. While the majority of women exhibited stable microbial cluster, a small subset experienced reshaping of their intestinal communities in response to pregnancy. When studying the gut microbiome during pregnancy, it is crucial to consider the individual variations in community structures within the healthy population. Additionally, attention should be given to the community characteristics of specific genera, as the same genera in different communities may have distinct effects on immunity.

## Data availability statement

The datasets presented in this study can be found in online repositories. The names of the repository/repositories and accession number(s) can be found at: BioProject, PRJNA997585.

## Ethics statement

The studies involving humans were approved by Institutional Review Board for Human Subjects Research at The First Affiliated Hospital of Jinan University. The studies were conducted in accordance with the local legislation and institutional requirements. The participants provided their written informed consent to participate in this study.

## Author contributions

HY: Methodology, Software, Visualization, Writing – original draft, Writing – review & editing. XL: Data curation, Investigation, Writing – review & editing. HL: Writing – review & editing. XT: Writing – review & editing. XX: Conceptualization, Funding acquisition, Project administration, Writing – review & editing.
